# Systematic Review: Cardiac Metastasis of Lingual Squamous Cell Carcinoma

**DOI:** 10.51894/001c.27297

**Published:** 2021-08-30

**Authors:** Connor C. Kerndt, Trevor A. Nessel, John A. Bills, Zaid J. Shareef, Alexander M. Balinski, Devin T. Mistry

**Affiliations:** 1 Department of Internal Medicine Spectrum Health/Michigan State University College of Human Medicine, Grand Rapids, Michigan, USA; 2 Michigan State University College of Osteopathic Medicine, East Lansing, Michigan, USA; 3 Oakland University William Beaumont School of Medicine, Rochester, Michigan, USA; 4 Department of Otolaryngology Metro Health Hospital-University of Michigan, Wyoming, Michigan, USA

**Keywords:** cancer, cardiac metastasis, squamous cell carcinoma, tongue

## Abstract

**INTRODUCTION:**

Lingual squamous cell carcinoma (LSCC) is an aggressive malignancy that carries significant mortality risk and the potential for cardiac metastasis. The authors performed a systematic review designed to characterize disease progression of LSCC cardiac metastasis by evaluating patient demographics, characteristics, management, and clinical outcomes.

**METHODS:**

Two authors independently screened articles in Embase, PubMed, and Cochrane Database of Systematic Reviews up until December 2019 for study eligibility. Demographic data, patient symptomatology, imaging findings, management strategies, and patient outcomes were obtained and analyzed. The Oxford Centre for Evidence Based Medicine (OCEBM) Levels of Evidence categorization was implemented to determine the quality of studies selected in this review.

**RESULTS:**

From this review, a total of 28 studies met inclusion criteria and received an OCEBM Level 4 evidence designation. Thirty-one patients were identified with cardiac metastasis from LSCC. Shortness of breath (29.0%) and chest pain (29.0%) were the most common presenting symptoms, and pericardial effusion (29.2%) and right ventricular outflow tract obstruction (25.0%) were the predominant echocardiogram findings. Cardiac metastases most often presented in the right ventricle (56.7%), followed by the left ventricle (43.3%). Palliative intervention (68.2%) or chemotherapy (40.9%) were typically implemented as treatments. All sample patients expired within one year of metastatic cancer diagnosis in cases that reported mortality outcomes.

**CONCLUSIONS:**

Patients presenting with shortness of breath, tachycardia, and a history of squamous cell carcinoma of the tongue may indicate evaluation for LSCC cardiac metastasis. Although LSCC cardiac metastases typically favor the right and left ventricles, they are not exclusive to these sites. Palliative care may be indicated as treatment due to high mortality and overall poor outcomes from current interventions.

## INTRODUCTION

Cancer of the oropharynx (i.e., part of the throat at the back of the mouth behind the oral cavity) is one of the most frequently diagnosed cancers worldwide, representing the seventh largest incidence burden of new cancer in men and fourteenth amongst women.^1–4^ Lingual squamous cell carcinoma (LSCC) accounts for approximately 3.0% of oropharyngeal carcinomas,^4^ typically affecting male smokers older than 45 years of age.^5,6^ Although LSCC is rare, global rates have been shown to increase from 0.4% to 3.3% during recent years, and there is an increasing incidence in the young female population.^7^

LSCC primarily confers metastatic risk to the lungs, heart, and bones, although it has demonstrated the ability to metastasize to nearly all organ systems.^3^ As a result, presentations of metastatic LSCC are exceptionally variable and contingent on the site of metastasis. It has also been estimated that metastatic LSCC will show cardiac involvement between 1.5% to 24.0% of all cases.^8^

Most cases of LSCC cardiac metastasis are detected post-mortem, although ante-mortem cases can be detected when symptomatic.^9^ Symptoms of cardiac metastasis are relatively non-specific, but can include fatigue, chest pain, orthopnea, and leg edema.^8^ To date, characterization of metastatic LSCC of the heart has been limited to case reports and case series.

### Purpose of Review

The purpose of this systematic review was to examine the patient demographics, characteristics, management, treatments, complications, and outcomes associated with LSCC cardiac metastases.

## METHODS

This systematic review was completed in 2020 using the *Preferred Reporting Systems for Systematic Reviews and Meta-Analysis* (PRISMA) guidelines.^10^ Preliminary searches were performed using data from PubMed, Embase, and Cochrane Library comprising studies dated through December 2019. The primary search included the keywords “tongue cancer”, “lingual cancer”, “buccal cancer”, and “metastasis”. A secondary search was done using the keywords “cardiac metastasis” and “tongue”.

**Figure 1.**
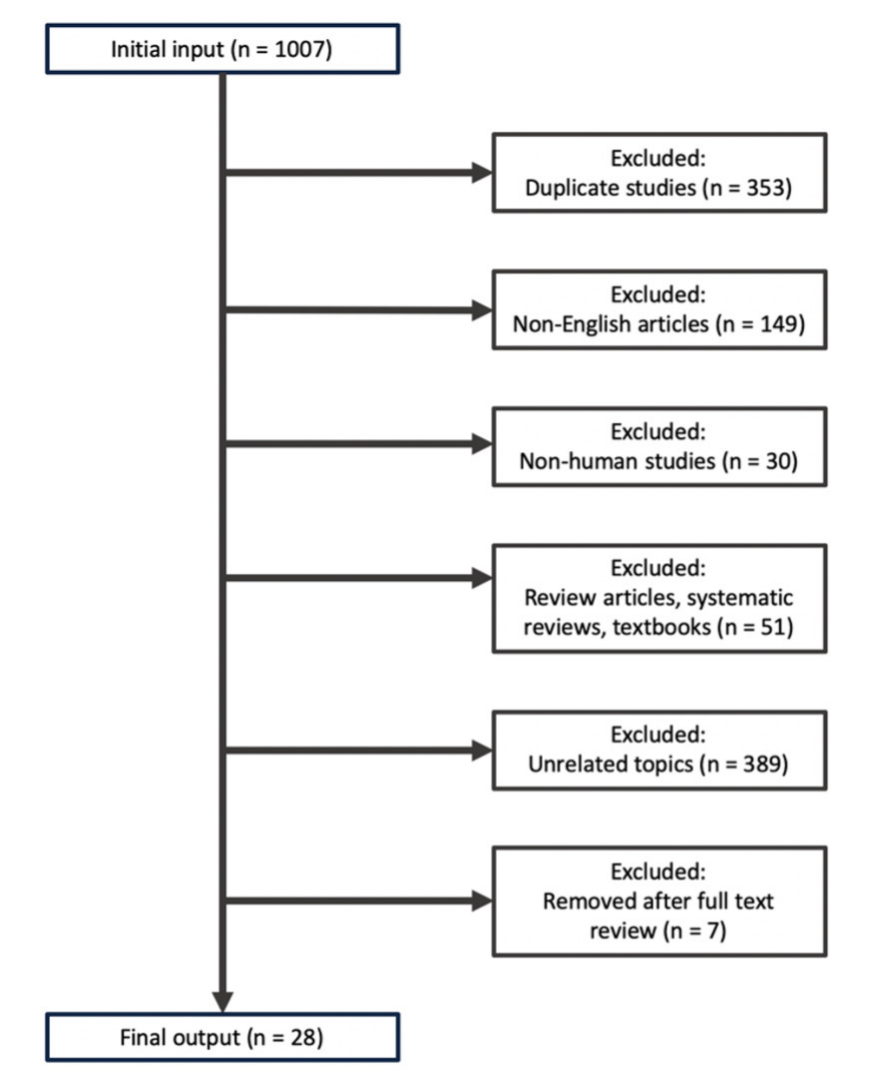
Literature selection criteria. Literature selection methodology was constructed using the *Preferred Reporting Items for Systematic Reviews and Meta-Analyses* (PRISMA).

The selected articles were combined to create a composite list of 1,007 studies to review. Studies that included review articles, textbooks, non-human subjects, non-English language, or unrelated topics were excluded. The inclusion criteria utilized in this systematic review are outlined in Figure 1.

### Study selection and data extraction

Each article within the composite list of studies was reviewed for inclusion by two independent authors (CK, TN). The titles and abstracts were screened for information regarding metastatic LSCC presentation, diagnosis, and management. Relevant articles were further examined via full text review and a finalized list was generated for in-depth analysis (Supplemental Table 1). A total of 31 cases of LSCC cardiac metastasis within 28 studies met inclusion criteria and were comprehensively reviewed (Supplemental Table 2).

All 28 selected studies had obtained an Oxford Centre for Evidence-Based Medicine (OCEBM) evidence categorization of Level 4 given their status as either a case report or case series. Relevant patient demographics, symptoms, LSCC history, clinical findings, treatment strategies, complications, and outcomes were collected. It was assumed death had occurred within six months if patients underwent palliative care.

## RESULTS

### Sample Demographics and Exposure

**Figure 2.**
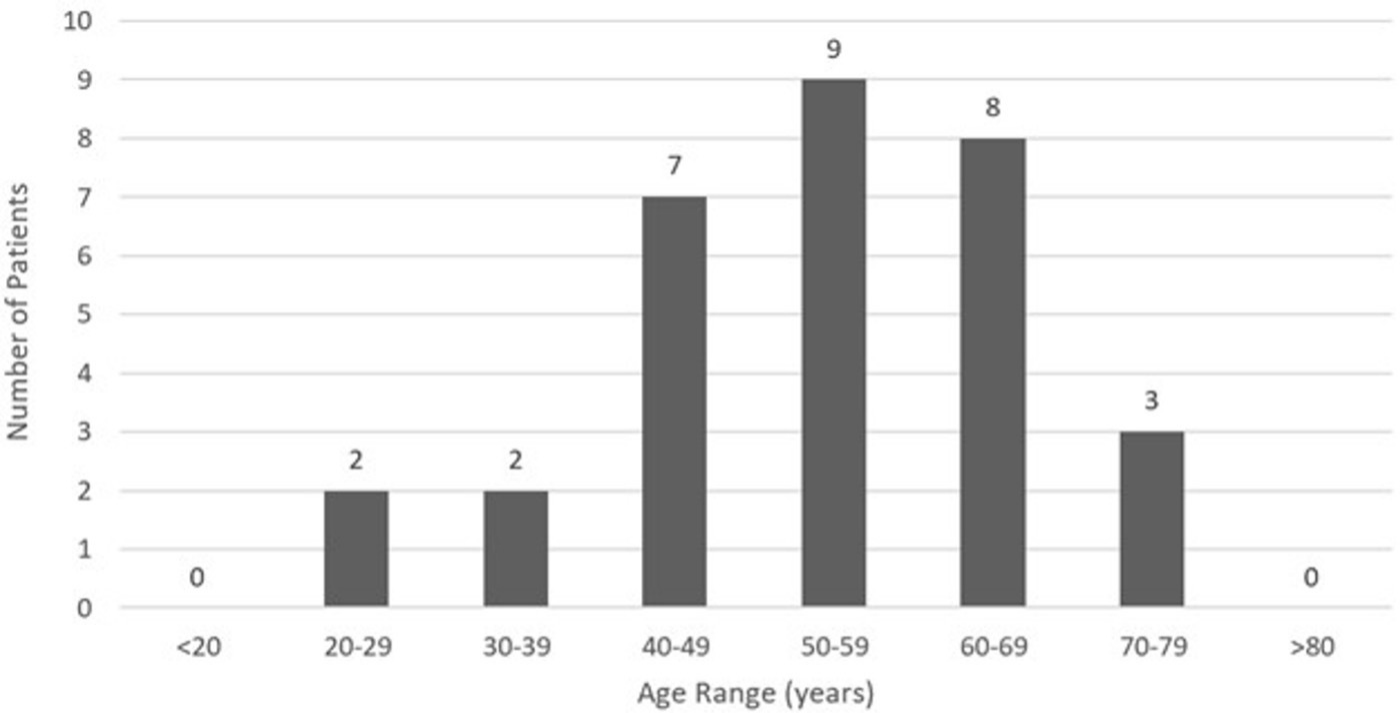
LSCC age distribution by decade of life.

An analysis of demographic characteristics of patients in selected articles included 19 (61.3%) males and 12 (38.7%) females with a mean age of 53.6 (SD = 12.9) years ranging from 23.0 - 77.0 years. LSCC cardiac metastasis presented primarily between the ages of 40 - 69 (Figure 2). Average time from primary cancer diagnosis to cardiac metastasis identification was 2.2 (SD = 2.6) years, ranging from 0.2 - 11.0 years. Ten (32.3%) patients reported significant tobacco exposure and five (16.1%) patients admitted to alcohol use.

### Presenting Symptoms and Physical Examination

**Figure 3:**
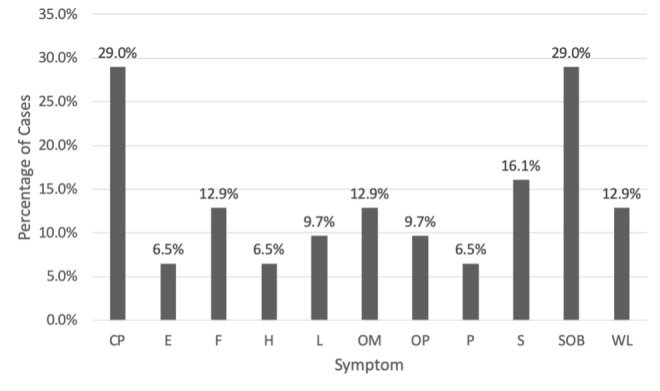
Symptomatology of LCCC Patients diagnosed with cardiac metastasis. Abbreviations: CP = chest pain, E = edema, F = fever, H = hemoptysis, L = lymphadenopathy, OM = oral mass, OP = oral pain, P = palpitations, S = syncope, SOB = shortness of breath, WL = weight loss.

Chest pain and shortness of breath were the most common causes of initial presentation with nine (29.0%) cases, followed by five (16.1%) cases with syncope, four (12.9%) cases with weight loss, four (12.9%) cases with fever, four (12.9%) cases with oral mass, three (9.7%) cases with lymphadenopathy (i.e., enlargement of lymph nodes > or = 1), and three (9.7%) cases with oral pain. Other symptoms included two (6.5%) patients with edema, two (6.5%) patients with hemoptysis (i.e., blood mixed in sputum), and two (6.5%) patients with palpitations. A complete outline of patient symptomatology is depicted in Figure 3.

The most common physical examination findings included four (12.9%) patients with hypotension, four (12.9%) patients with fever, three (9.7%) patients with tachycardia, and three (9.7%) patients with cardiac murmur.

### Cardiac Evaluation

Electrocardiogram (ECG) testing was reported in 19 (61.3%) patients, which found the most common abnormality to be ST-segment elevation in 12 (63.2%) patients (Supplemental Table 3). Other commonly reported ECG findings included arrhythmia in six (31.6%) patients, bundle branch block in four (21.1%) patients, t-wave inversion in four (21.1%) patients, and tachycardia in three (15.8%) patients. Troponin testing was reported in 31 patients with positive troponin elevations occurring in five (16.1%) cases.

### Imaging Modalities and Findings

**Figure 4:**
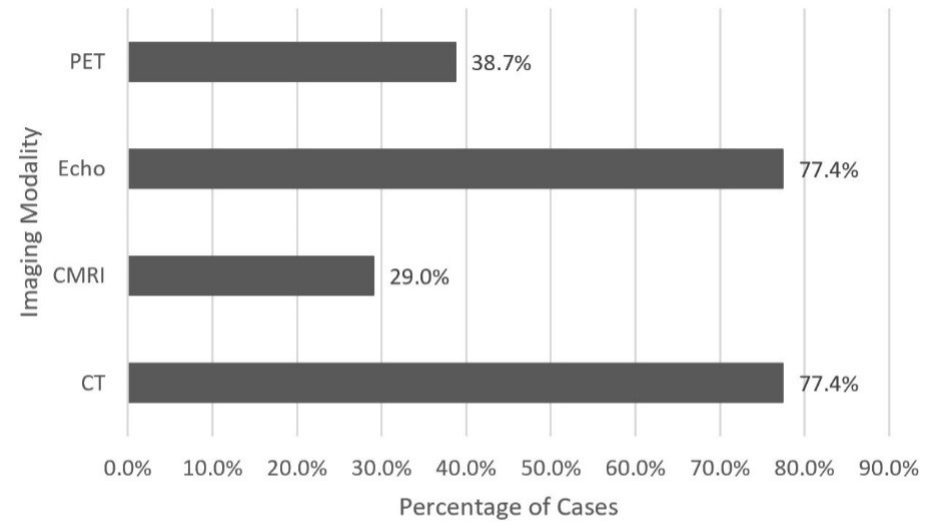
Utilization of diagnostic modalities. Four major imaging modalities were used to assist with the diagnosis of cardiac metastasis from LSCC. Abbreviations: CMRI = cardiac magnetic resonance imaging; CT = computed tomography; Echo = echocardiogram; PET = positron emission tomography.

The most utilized imaging modalities were echocardiogram and computed tomography (CT) both occurring in 24 (77.4%) cases, followed by positron emission tomography (PET) in 12 (38.7%) cases, and cardiac magnetic resonance imaging (CMRI) in nine (29.0%) cases (Figure 4). On echocardiogram, the most common finding was pericardial effusion occurring in seven (29.2%) cases, followed by six (25.0%) cases with right ventricular outflow tract obstruction, three (12.5%) cases with valvular dysfunction, and two (8.3%) cases with wall motion abnormality (i.e., kinetic alterations in the cardiac wall motion taking place during the cardiac cycle) (Supplemental Table 3).

Of the patients with reported valvular dysfunction, two (66.6%) cases had impaired function of the pulmonic valve and one (33.3%) case had impaired tricuspid valve function. In the 12 patients who underwent PET scan, extra-cardiac uptake was most reported in the lung with five (41.7%) reported cases, followed by two (16.7%) cases in the bone, two (16.7%) cases in the liver, and one (8.3%) case in the muscle. The number of extra-cardiac foci ranged from 0.0 - 7.0 sites with an average of 1.8 (SD = 1.7) sites.

### Location of Cardiac Metastasis

Out of 31 selected cases, 27 disclosed the number of cardiac metastases. Number of distinct cardiac metastases ranged from 1.0 - 4.0, with an average of 1.5 (SD = 0.8) metastases. Most cases presented with one individual mass in 17 (63.0%) cases, followed by two masses in 8 (29.6%) cases, and three or more masses in 2 (7.4%) cases.

The locations of metastases were distinctly specified in 30 of 31 cases. Right-sided metastases were most common with 23 (76.7%) instances, followed by 15 (50.0%) instances of left-sided metastases, and 11 (36.7%) instances of septal metastases. The most common location of metastasis was the right ventricle with 17 (56.7%) instances, followed by the left ventricle in 13 (43.3%) instances, interventricular septum in nine (30.0%) instances, and right atrium in six (20.0%) instances. This analysis also revealed eight (26.7%) patients with pericardial metastasis. Locational distinctions are further defined in Supplemental Table 2 and Supplemental Table 4.

### Patient Management

Primary LSCC treatment strategies were discussed in 29 (93.5%) of 31 total cases. The most frequently reported primary treatment was surgery in 25 (86.2%) cases, followed closely by radiotherapy in 23 (79.3%) cases, and chemotherapy in 13 (44.8%) cases. The most common surgical intervention was neck dissection in eight (32.0%) cases, followed by partial glossectomy (i.e., removal of tongue tissue) in six (24.0%) cases, and hemi-mandibulectomy (i.e., removal of part of or all of the hemimandible, or one side or half of the mandible) in two (8.0%) cases.

Treatment strategies for metastatic lesions were described in 22 (71.0%) of 31 cases which included chemotherapy, surgical intervention, radiotherapy, immunotherapy, and palliative treatment. Given the poor prognosis, the most elected therapy was palliative treatment in 15 (68.2%) instances. Chemotherapy was the next most common intervention in nine (40.9%) instances, followed by surgery in four (18.2%) instances, radiotherapy in three (13.6%) instances, and immunotherapy in one (4.5%) instance.

Of those treated with chemotherapy, the most used agents were cisplatin in three (33.3%) cases and 5-fluorouracil in three (33.3%) cases. Other chemotherapeutics included bleomycin, etoposide, and methotrexate. Of note, immunotherapy with pembrolizumab was utilized in only one (4.5%) case. Individual treatments are further outlined in Supplemental Table 2.

### Patient Outcomes

Twenty-two total cases (70.9%) reported outcome results, of which none showed remission of LSCC cardiac metastasis. Death was reported in 20 (90.9%) cases with two (9.1%) cases reporting palliative care and assumed death in less than six months. Of the 14 cases to report death within a known period, 9 (64.3%) reported death within one month of LSCC cardiac metastasis diagnosis.

Cause of death was listed in 16 cases which included eight (50.0%) deaths from cardiac metastasis, two (12.5%) deaths from sepsis, two (12.5%) deaths from arrhythmia/sudden cardiac death, one (6.3%) death from heart failure, one (6.3%) death from hemoptysis, one (6.3%) death from death in sleep, and one (6.3%) death from tumor embolism. Four (25.0%) cases had cardiac-related deaths, while 12 (75.0%) cases had tumor-specific or non-cardiac deaths.

## DISCUSSION

Primary squamous cell carcinoma (SCC) accounts for more than 90% of cancer diagnoses within the head and neck region and is the sixth most common cancer overall by incidence.^11^ Classically, LSCC displays a predilection for the elderly male population,^12^ although it can present more aggressively in younger individuals with higher rates of metastasis and mortality.^13^ Recent research also suggests that young Caucasian females are demonstrating an overall increase in disease incidence.^7^ In this study, the majority of patients (51.6%) were males aged 40-69 years old, consistent with the typical presentation of LSCC.

In addition to age and sex, modifiable factors such as tobacco and alcohol exposure carry significant independent and synergistic risks for LSCC.^14,15^ Exposure to viral infections such as Epstein-Barr virus (EBV) and human papilloma virus (HPV) have also been associated with LSCC.^16,17^ In this systematic review, most cases failed to describe previous history of alcohol or tobacco use and no cases reported previous EBV or HPV exposure.

Cardiac metastasis of LSCC is typically diagnosed post-mortem on autopsy due to limited specific clinical manifestations.^18^ When diagnosed ante-mortem, patients may present with nonspecific cardiac symptoms such as chest pain, shortness of breath, and palpitations.^19^ In this systematic review, patients commonly demonstrated nonspecific cardiac symptoms such as chest pain, shortness of breath, syncope, and weight loss. Of these symptoms, chest pain and shortness of breath were the most prevalent (29.0% of cases), followed by syncope, then weight loss. Common physical exam findings were generally nonspecific (e.g., hypotension, fever, tachycardia, and cardiac murmur) in the selected articles.

Primary SCC originating from the head and neck with cardiac metastasis is extremely rare, as most cardiac neoplasms occur secondarily from areas such as the breast, lung, and esophagus.^20^ When present, cardiac metastasis most frequently occurs in the pericardium, followed by the myocardium, epicardium, endocardium, and intracavitary region.^21^

In this systematic review, right-sided cardiac metastasis was more common than left-sided metastasis, and ventricular involvement was more common than atrial involvement. The examined cases demonstrated a higher incidence of intracardiac tumors compared to pericardial tumors. Possible explanations for these findings relate to the hematogenous spread of squamous cell neoplasms through the coronary arteries, direct contiguous extension, and retrograde lymphatic flow.^22^

Since symptomatic presentations of LSCC cardiac metastasis can be highly variable, imaging studies are needed to assess the extent of cardiac involvement. First-line imaging for cardiac metastasis of LSCC begins with an echocardiogram due to its wide availability and lack of radiation.^23^ When there are no contraindications, contrast-enhanced CT scans are the diagnostic imaging modality of choice, as non-contrast CT scans may fail to identify small myocardial masses.^24^ If further imaging is indicated due to inconclusive CT results, CMRI is considered the most definitive imaging modality for evaluation of myocardial metastasis and delineation of intracardiac tumor thrombi.^23^

Cervical lymph node involvement is a strong prognostic factor when delineating patient outcomes for LSCC.^25^ Five-year survival rates for patients with oral SCC lymph node metastasis are low at 25-40%, which contrasts with a 90% survival rate for individuals without lymph node metastasis.^12^ Metastases to the heart further worsen this prognosis with a median survival of approximately 3.5 months without treatment.^26–28^ Palliative care is typically indicated for LSCC cardiac metastasis patients, as most cases present with poorly prognostic advanced cardiac SCC metastasis.^26^

During this review, no example of remission or significant survival was demonstrated in patients with cardiac metastasis of LSCC. Many cases resulted in cardiac-related death within 30 days of cardiac metastasis presentation and five deaths occurred upon initial admission for metastatic evaluation. These findings suggest that palliation may be the most appropriate treatment strategy for patients with LSCC cardiac metastasis given the poor prognosis with current treatment strategies.

### Review Limitations

This systematic review had several limitations. First, only 31 cases of cardiac metastasis from LSCC met inclusion criteria which limits the power of this study. Second, LSCC cardiac metastasis may have been underreported in the medical literature due to missed diagnoses from lack of symptomatology or post-mortem follow-up. Third, the results of this study may not be completely representative of all documented cases of LSCC cardiac metastasis due to the omission of many non-English case reports and case series.

## CONCLUSIONS

Cardiac metastasis from primary LSCC demonstrates a dangerous, uncommon presentation of malignancy. Clinical suspicion for LSCC cardiac metastasis should arise in patients with new onset chest pain and shortness of breath in the setting of prior diagnosis of LSCC. Prior tobacco and alcohol use should generate additional diagnostic speculation in these patients.

In the setting of previously known disease, advanced imaging such as echocardiogram, CT, and CMRI may prove useful for identification of disease progression. LSCC cardiac metastases typically favor the right and left ventricles, but are not exclusive to these sites. Due to the poor prognostic implications of LSCC cardiac metastasis, myocardial biopsy is unlikely to alter patient management and palliative discussions should be considered.

### Conflict of Interest

None

## Supplementary Tables

**Supplemental Table 1.** Level of evidence and conclusion of studies.

**Authors**	**Study Year**	**LOE (1a-5)**	**Study Design**	**Subjects**	**Authors’ Conclusions**
Tandon^20^	2019	4	CR	1	Patients with current malignancies in addition to cardiac symptoms and/or ECG changes should warrant investigation for possible cardiac metastasis. Different imaging modalities such as CMRI, PET-CT, and echocardiography play an important role in diagnosis and management.
Nagata^29^	2012	4	CR	1	Cardiac metastasis from LSCC is typically a difficult diagnosis due to the lack of clinical findings in the early stages. However, as the metastases become more advanced, clinical signs may occur, resulting in a variety of cardiac pathologies. Many of the cardiac sequelae result in ECG changes, emphasizing the importance of ECG testing in these patients.
Onwuchekwa^30^	2012	4	CS	2	These cases show the importance of evaluating the heart with echocardiography to assess for cardiac metastasis in patients with head and neck cancer and confirmed metastatic disease.
Browning^31^	2015	4	CR	1	It may be necessary to evaluate patients with lingual malignancies with imaging to rule out metastases. Lingual squamous cell carcinoma metastases are commonly asymptomatic, and thus, imaging is important in establishing the diagnosis.
McKeag^32^	2013	4	CR	1	This case shows that cardiac metastasis from LSCC can present without symptoms or characteristic metastasis examination findings such as lymphadenopathy.
Hans^33^	2009	4	CR	1	Clinicians may consider the potential for cardiac metastases in patients with a history of head and neck malignancies who display new-onset cardiovascular symptoms. These patients typically have a very poor prognosis.
Kumar^34^	2018	4	CR	1	Clinicians should consider cardiac metastasis and hypercalcemia-induced rhythm disturbances in patients with a history of LSCC malignancy or current LSCC malignancy and concurrent ECG changes. It may be necessary to check calcium levels and treat the patient if levels are abnormal.
Nanda^35^	2019	4	CR	1	This case shows the importance of an initial echocardiogram in addition to the integration of PET and CT scans to detect LSCC cardiac metastasis.
Werbel^36^	1985	4	CR	1	This case demonstrates the importance of using echocardiography in patients with LSCC and atypical angina and/or a new heart murmur. Echocardiography is a good initial step to look for cardiac metastases.
Puranik^37^	2014	4	CS	2	In asymptomatic patients with a history of cancer, whole body dual imaging with PET-CT can show undiagnosed metastatic sites.
Makhija^38^	2009	4	CR	1	This case report stresses the lack of appropriate screening protocols for patients with LSCC and possible cardiac metastases. The report recommends a transesophageal echocardiogram as the best initial screen, as it is a sensitive test.
Wadler^39^	1985	4	CR	1	This case report emphasizes that there are many other factors besides morphology and anatomic extent that can be used to determine LSCC prognosis.
Shafiq^40^	2019	4	CR	1	This case report shows that the lack of symptoms in cardiac metastasis from LSCC can result in delayed cardiac imaging and subsequently delayed diagnosis.
Dredla^41^	2014	4	CR	1	Patients with a history of cancer who have a cerebrovascular accident should have cardiac imaging to rule out a cardiac cause, such as cardiac metastases.
Yadav^42^	2014	4	CR	1	This case highlights the importance of an ECG evaluation in patients with a cancer diagnosis. This can provide diagnostic clues for potential cardiac metastases. Diagnostic confirmation can be done using multiple imaging modalities, such as echocardiography, CT scan, and CMRI.
Zatuchni^43^	1981	4	CS	2	LSCC cardiac metastasis can result in myocardial or pericardial injury, shown by ST-elevations on ECG. ST-elevation may be a potential sign of cardiac metastasis.
Shafiq^44^	2019	4	CR	1	This case emphasizes the importance of the clinician maintaining a high index of suspicion to identify patients with metastatic LSCC. A high risk patient may need surveillance imaging with echocardiography, CMRI, and/or PET-CT.
Kim^45^	2019	4	CR	1	LSCC patients with cardiac symptoms may benefit from a multimodal approach consisting of PET-CT, CMRI, echocardiography, and ECG. These imaging modalities are used to confirm the diagnosis and establish the location and extent of disease, which can guide management.
Demir^46^	2018	4	CR	1	This case shows the importance of keeping myocardial metastases on the differential diagnosis when patients with known malignancies present with ECG changes. Cardiac metastases can sometimes present like an acute myocardial infarction on ECG testing.
Chua^47^	2017	4	CR	1	LSCC is more common in Asian countries. It is always important to consider metastasis of LSCC to the heart and other organs, especially if patients with known LSCC present with cardiac symptoms.
Duband^48^	2011	4	CR	1	Clinicians may consider an initial cardiac evaluation and serial follow-up ECGs in patients with SCC of the base of the tongue.
Ito^49^	2007	4	CR	1	Sudden death in patients with LSCC should prompt clinicians to consider the potential of cardiac metastases.
Rivkin^50^	1999	4	CR	1	Patients with cancer who develop new cardiac symptoms should prompt the clinician to evaluate for potential cardiac metastases. Evaluation should consist of echocardiography and/or CMRI. Treatments are typically only palliative, as cardiac metastases possess a poor prognosis.
Matsuyama^51^	1963	4	CR	1	This case reports a rare finding of widespread LSCC metastasis with cardiac metastases isolated to the myocardium. This is a rare finding since most metastases affect the pericardium.
Malekzadeh^19^	2017	4	CR	1	Patients with a history of head and neck malignancy who present with cardiac symptoms may prompt the clinician to evaluate for cardiac metastases.
Cabot^52^	1971	4	CR	1	There are often no clinical signs of primary LSCC or metastatic LSCC to the heart, making the ante-mortem diagnosis very difficult.
Reddy^53^	2014	4	CR	1	Management of tumor-induced acute coronary occlusion is similar to acute coronary syndrome occlusion, utilizing coronary angiography and percutaneous coronary intervention.
Ewald^54^	1997	4	CR	1	Patients with a history of malignancy and unexplained arrhythmias or ECG changes may prompt the clinician to consider cardiac metastases on the differential diagnosis. Cardiac metastasis may present similarly to many types of cardiac pathology.

Abbreviations: CMRI = cardiac magnetic resonance imaging; CR = case report; CS = case series; CT = computed tomography; ECG = electrocardiogram; LSCC = lingual squamous cell carcinoma; PET = positron emission tomography; PET-CT = positron emission tomography-computed tomography; SCC = squamous cell carcinoma.

**Supplemental Table 2.** Detailed Patient Information.

**Age/Sex**	**Primary Location**	**Signs & Symptoms**	**Metastasis Locations**	**First Identifying Imaging**	**Primary** **Treatment**	**Metastasis Treatment**	**Outcomes**
M 59	Right tongue mass	Fever	LA & pericardium	CT & Echo	Partial glossectomy, right RND, CTX	Cardiac surgery	Death 3 weeks after surgery
F 45	Tongue	Syncope & dyspnea	RV, LV, IVS	CTA & Echo	Partial right glossectomy, RND, RT.	RT (brain)	NR
F 36	Tongue	Palpitations & SOB	LV	CT & Echo	CTX-RT, partial left glossectomy, and left neck LN dissection	RT	Death 2 months after
M 50	Base of tongue	Odynophagia, otalgia, dysphonia, WL, tongue and neck pain	Apex of RV	PET-CT	2 months radiation, total glossectomy & bilateral RND	None	NR
M 77	NR	Recurrent syncope	RA & RV infiltration of myocardium	Echo	None	None	Death 28 days after diagnosis
M 54	Tongue base	Dyspnea, LE edema, & hemoptysis	RV	CT & Echo	Glossectomy, left RND, cisplatin, 5-FU	None	Death
F 23	Tongue	CP, Right arm paresthesias, cough, SOB	LV & RV infiltration of myocardium	Echo	Surgical resection with clear margins	None	NR
M 28	Tongue	Recurrent syncope	RA, LV, IVS	Echo	Hemimandibulectomy & post-operative RT	Pacemaker implantation	Death 5 days after pacemaker placement
M 47	Tongue	Dizziness, SOB, chest tightness, night sweats	RA, RV, pulmonary artery	CT chest	Surgical excision with CTX-RT	None	Patient lost to follow-up
F 61	Base of tongue	CP, palpitations	RA & pericardium	Echo	Hemiglossectomy with excisional biopsy of digastric LN. Neck RT	None	Death 7 weeks after cardiac diagnosis
F 32	Right. lateral tongue	Nasal swelling	LV & IVS	PET-CT scan	Wide excision & right lateral neck dissection	CTX	Follow-up CT stable
M 46	Right vallecula	N/A (Follow-up)	RV	Endoscopy	CTX-RT	CTX	Patient lost to follow-up
F 66	Tongue	Anginal symptoms	RV	Coronary angiogram	Radical resection	None	NR
M 57	Anterior tongue	Fever, dyspnea	Epicardium, myocardium, endocardium	Echo	Partial glossectomy	CTX	Death 14 days after admission from sepsis
M 43	Tongue	N/A (Follow-up)	Apex of LV & RV	CT & Echo	CTX-RT	CTX	Death due to cardiac metastasis
M 61	Left tongue base	Cerebral infarction symptoms	LAA and pulmonary vein	CT & Echo	CTX-RT	None	NR
M 76	Tongue	Pneumonia	RV, LV	CT chest	Partial glossectomy, multiple neck dissection, CTX	Palliative care	Died in one month
M 61	Tongue	CP, dyspnea	IVS and pericardium	99mTC scan	RAD	None	Death
M 62	Tongue	Malaise	Diffuse myocardium	99mTC scan	NS	None	Death
M 43	Right tongue	N/A (Follow-up)	RV, LV	CT chest	Right neck dissection & tongue resection, free flap reconstruction, CTX-RT	CTX-RT	Death
F 46	Left lateral tongue	Left arm, ear, and throat pain	LV, pericardium	CT	Hemiglossectomy, bilateral neck dissection, CTX-RT	CTX-RT	NR
M 59	Tongue	Chest discomfort	RV	Echo	Resection, RT	None	Death
M 63	Tongue	Dyspnea	RV	Echo	Resection and reconstruction	None	NR
F 57	Tongue	Death	RA, AV sulcus	Autopsy	Hemiglossectomy, bilateral cervical lymphadenectomy, CTX-RT	None	Death
M 66	Right tongue into oropharynx	Death	LV, IVS	Autopsy	Total glossolaryngectomy and dissection LN bilaterally	None	Death
M 57	Right tongue base	Lower extremity edema	RV	Echo, ECG, CMRI, CT	Local excision with post-operative RT to lesion and neck	CTX	Death
F 48	Anterior tongue	CP, dyspnea, hemoptysis, anorexia.	Diffuse myocardium, RA, LV, aortic valve	NR	Anterior tongue glossectomy, excision of submandibular LN	None	Death
F 58	Tongue	CP	NR	CT	Hemiglossectomy and RT	Palliative CTX and immunotherapy	Death
F 70	Tongue	Fever & chills	Diffuse pericardium	CT	Left hemiglossectomy, RND	Thoracotomy	Death
F 52	Tongue	Dull CP	RV, LV, IVS	PET	Hemiglossectomy and RND	Pericardial window and palliative care	Life expectancy < 3 months
M 60	Base of tongue	Malnutrition, pneumonia	Anterior wall of RV & pulmonic valve	Echo	RT and a composite tongue-jaw-neck resection	Palliative care	Death

Abbreviations: 5-FU = 5-fluorouracil; 99mTC = Technetium 99; CMRI = cardiac magnetic resonance imaging; CP = chest pain; CT = computed tomography; CTA = computed tomography angiography; CTX = chemotherapy; CTX-RT = chemotherapy/radiation; Echo = echocardiogram; IVS = interventricular septum; LA = left atrium; LAA = left atrial appendage; LE = lower extremity; LN = lymph node; LV = left ventricle; MRI = magnetic resonance imaging; NR = not recorded; NS = non-surgical; PET = positron emission tomography; PET-CT = positron emission tomography-computed tomography; RA = right atrium; RND = radical neck dissection; RT = radiation therapy; RV = right ventricle; SOB = shortness of breath; WL = weight loss.

**Supplemental Table 3.** Clinical patient characteristics.

**Characteristics**	**Data**
No. of subjects	31
Male, n (%)	19 (61.3)
Female, n (%)	12 (38.7)
Mean age of diagnosis, n years (range)	53.6 (23.0-77.0)
Mean primary to metastasis diagnosis, n years (range)	2.2 (0.2-11.0)
**Presenting symptoms, n (%)**	
Chest pain	9 (29.0)
Shortness of breath	9 (29.0)
Syncope	5 (16.1)
Weight loss	4 (12.9)
Fever	4 (12.9)
Oral mass	4 (12.9)
Lymphadenopathy	3 (9.7)
Oral pain	3 (9.7)
Edema	2 (6.5)
Hemoptysis	2 (6.5)
Palpitations	2 (6.5)
**Physical exam findings, n (%)**	
Hypotension	4 (12.9)
Fever	4 (12.9)
Cardiac murmur	3 (9.7)
Tachycardia	3 (9.7)
**Diagnostic findings, n (%)**	
*Electrocardiography*	19 (61.3)
ST elevation	12 (63.2)
Arrhythmia	6 (31.6)
Bundle branch block	4 (21.1)
T-wave inversion	4 (21.1)
*Echocardiography*	24 (77.4)
Pericardial effusion	7 (29.2)
Right ventricular outflow tract obstruction	6 (25.0)
Valvular dysfunction	3 (12.5)
Wall motion abnormality	2 (8.3)

**Supplemental Table 4.** Tumor location.

**Characteristics**	**Data**
**Primary tumor site, n (%)**	
Tongue	31 (100.0)
**Cardiac metastases, n (%) (1 case omitted)**	30 (96.8)
Right-sided metastasis	23 (76.7)
Left-sided metastasis	15 (50.0)
Septal metastasis	11 (36.7)
Pericardial metastasis	8 (26.7)
*Specific location*	
Right ventricle	17 (56.7)
Left ventricle	13 (43.3)
Interventricular septum	9 (30.0)
Right atrium	6 (20.0)
Left atrium	2 (6.7)
